# MALDI-TOF-MS analysis in discovery and identification of serum proteomic patterns of ovarian cancer

**DOI:** 10.1186/s12885-017-3467-2

**Published:** 2017-07-06

**Authors:** Agata Swiatly, Agnieszka Horala, Joanna Hajduk, Jan Matysiak, Ewa Nowak-Markwitz, Zenon J. Kokot

**Affiliations:** 1 Department of Inorganic and Analytical Chemistry, Poznan University of Medical Sciences, ul. Grunwaldzka 6, 60-780 Poznań, Poland; 2 Gynecologic Oncology Department, Poznan University of Medical Sciences, ul. Polna 33, 60-535 Poznań, Poland

**Keywords:** Epithelial ovarian cancer, Ovarian cancer, Biomarkers, MALDI-TOF, Protein-peptide profiling

## Abstract

**Background:**

Due to high mortality and lack of efficient screening, new tools for ovarian cancer (OC) diagnosis are urgently needed. To broaden the knowledge on the pathological processes that occur during ovarian cancer tumorigenesis, protein-peptide profiling was proposed.

**Methods:**

Serum proteomic patterns in samples from OC patients were obtained using matrix-assisted laser desorption/ionization time-of-flight mass spectrometry (MALDI-TOF). Eighty nine serum samples (44 ovarian cancer and 45 healthy controls) were pretreated using solid-phase extraction method. Next, a classification model with the most discriminative factors was identified using chemometric algorithms. Finally, the results were verified by external validation on an independent test set of samples.

**Results:**

Main outcome of this study was an identification of potential OC biomarkers by applying liquid chromatography coupled with tandem mass spectrometry. Application of this novel strategy enabled the identification of four potential OC serum biomarkers (complement C3, kininogen-1, inter-alpha-trypsin inhibitor heavy chain H4, and transthyretin). The role of these proteins was discussed in relation to OC pathomechanism.

**Conclusions:**

The study results may contribute to the development of clinically useful multi-component diagnostic tools in OC. In addition, identifying a novel panel of discriminative proteins could provide a new insight into complex signaling and functional networks associated with this multifactorial disease.

**Electronic supplementary material:**

The online version of this article (doi:10.1186/s12885-017-3467-2) contains supplementary material, which is available to authorized users.

## Background

Ovarian cancer (OC) is one of the leading causes of death among all gynecological malignancies [1]. As there are no early specific symptoms, OC is diagnosed in advanced clinical stages in more than 70% cases when, despite appropriate treatment, 5-year survival rate drops to 30% [2]. Early diagnosis improves treatment outcomes and also dramatically reduces mortality rate [3]. However, adequate diagnostic methods are lacking and therefore novel technologies that would allow early detection of OC are urgently needed.

Serum measurement of cancer antigen 125 (CA125) and transvaginal ultrasound examination have become the most widely used methods in OC diagnosis [4]. Nonetheless, they are characterized by low specificity, especially in early stage cancer and in women before menopause [5]. Extensive efforts to identify other OC biomarkers led to the discovery of human epididymis protein 4 (HE4). Usefulness of HE4 in diagnosis of OC has been widely explored [6–8]. As single cancer biomarkers were insufficient to detect a tumor in its early stages, many studies focused on the development of multi-marker serum panels [3, 9]. Food and Drug Administration (FDA) cleared for use two multiple biomarker tests: Risk of Ovarian Malignancy Algorithm (ROMA) and OVA1 - a multivariate index assay (MIA). ROMA combines serum CA125 and HE4 levels with menopausal status. This predictive probability algorithm allows for classifying patients into high and low risk OC groups [10]. The OVA1 test is a proprietary algorithm that combines serum concentrations of five markers (CA125, apolipoprotein A-1, β2-microglobulin, transthyretin and transferrin) and calculates a malignancy risk index score [9].

Despite the use of multi-marker diagnostic strategies, early detection of OC remains far from satisfactory. Thus, new strategies based on novel methodology such as proteomic research have been employed in OC research [11]. In recent years, untargeted proteomics, such as protein-peptide profiling, has emerged as an interesting tool for clinical diagnostics [12–14]. Identification of distinctive pattern of protein expression is a promising strategy for understanding molecular alterations during pathological processes [15]. Subsequently, the obtained information could be useful in detection of specific biomarkers and could increase the efficacy of early diagnosis [16]. One of the most frequently used tools in proteomic research (besides ESI - electrospray ionization) is matrix-assisted laser desorption/ionization time-of-flight mass spectrometry (MALDI-TOF MS) [17]. MALDI-TOF instruments have been reported sensitive and robust for clinical trials [18]. However, in the studies based on mass spectrometry analyses of complex biological samples like blood, serum or plasma, application of enrichment strategies seems to be necessary for generating good quality mass spectra [19]. Highly abundant proteins, as well as the presence of lipids and salts, mask other low abundant compounds, including cancer-related biomarkers [20]. Therefore, many different strategies have been proposed to pretreat plasma or serum samples. Currently, MALDI-TOF MS combined with ZipTip micropipette tips based on solid phase extraction proved successful. Moreover, several studies explored robustness and reliability of this methodology in protein-peptide profiling [20, 21].

The aim of this study was to characterize MALDI-TOF-MS-based serum proteomic patterns of OC and to identify differences in those patterns between OC samples and healthy control group. As far as we know, the combination of solid phase extraction pretreatment with MALDI-TOF-MS in OC research was presented for the first time. The MS data obtained were further processed and analyzed with advanced chemometric tools. A classification model containing the most discriminative peaks was calculated based on the obtained spectra and verified using an independent test set. Potential OC serum biomarkers were identified using nano-liquid chromatography (nano-LC) coupled with MALDI-TOF-MS/MS, since they might provide a new insight into the multifactorial processes that occur during OC tumorigenesis. To the best of our knowledge this is the first study in which novel OC protein patterns have been both discovered and identified based on MALDI-TOF MS techniques.

## Methods

### Characteristics of the study groups

Blood samples were collected from 89 patients operated in Gynecologic Oncology Department of Poznan University of Medical Sciences, Poland, on the day before surgery, between August 2014 and December 2015. Blood samples were incubated for 30 min at room temperature for clotting and centrifuged for 15 min at 4000 rpm. The resulting sera were isolated and stored at −80 °C until analysis. All serum samples were handled using the same laboratory equipment and stored in the same type of plastic vials and boxes. Based on histopathological result the patients were divided into two groups: OC (including borderline ovarian tumors) (*N* = 44) and no pathology of the ovaries - further referred to as “control group” (*N* = 45). The control group consisted of patients operated (hysterectomy with bilateral salpingoophorectomy) due to reasons other than ovarian tumors and in which the final histopathological examination confirmed no existing ovarian pathology. All participants were after overnight fasting. The patients were selected according to the following exclusion criteria: other than epithelial OC, other cancers currently or in anamnesis, chronic metabolic diseases (diabetes, dyslipidemia), previous or current cancer treatment (radiotherapy, chemotherapy, hormonal therapy), relevant concomitant medication (anti-diabetic agents, statins, hormonal replacement therapy, oral contraception. Additionally two markers, CA124 and HE4, were measured in the OC group with an electrochemiluminescence immunoassay (Roche Diagnostics, Indianapolis, IN, USA). Detailed characterization of the studied groups, including demographic and clinical profiles, is presented in Table 1 and Additional file 1 Table S1. The project was approved by the Bioethics Committee of Poznan University of Medical Sciences, Poland (Decision No. 165/16).

**Table 1:** Study group characteristics

Patient group	Number of samples	Median age (min-max)	Median BMI (min-max)	% of postmenopausal	Average concentration of CA125 (U/mL)	Average concentration of HE4 (pmol/L)
OC training set - Type I OC * borderline - Type II	33 10 *5 23	57 (36–72)	26.81 (17.29–38.37)	23 (70%)	2381.29	1025.10
OC test set - Type I OC * borderline - Type II	11 3 *1 8	65 (32–78)	24.75 (22.27–31.62)	9 (82%)	2177.67	1261.00
Control training set	33	58 (19–73)	26.06 (21.15–40.06)	22 (67%)	not determined	not determined
Control test set	12	55 (31–63)	27.56 (22.43–35.70)	7 (58%)	not determined	not determined

### Serum samples pretreatment

Each sample was diluted in 0.1% trifluoroacetic acid (TFA) in water (1:5). In order to desalt and concentrate the samples, solid phase extraction method based on ZipTip C18 pipette tips was used according to the manufacturer’s protocol (Millipore, Bedford, MA, USA). The tips were conditioned with acetonitrile (ACN) and 0.1% TFA. The prepared samples were loaded onto the tips and the peptides were bound. After washing with 0.1% TFA, sample fractions were eluted using 50% ACN solution in 0.1% TFA.

### MALDI-TOF-MS protein and peptide profiling

Each eluent sample was mixed with matrix solution of α-cyano-4-hydroxycinnamic acid (0.3 g/L HCCA in a solution containing 2:1 ethanol:acetone, *v*/v) at the ratio of 1:10. One microliter of the sample/matrix solution was spotted onto the MALDI target (AnchorChip 800 μm, Bruker Daltonics, Bremen, Germany) and left to crystallize at room temperature. The samples from both study groups were analyzed in a random order and the disease status of the women was blinded to minimize variability and systematic errors. UltrafleXtreme MALDI-TOF/TOF mass spectrometer (Bruker Daltonics, Bremen, Germany) was used to perform MS analyses in the linear positive mode. Positively charged ions were detected in the m/z range of 1000–10,000 Da and 2000 shots were accumulated per one spectrum. The MS spectra were externally calibrated with the mixture of Peptide Calibration Standard and Protein Calibration Standard I at the ratio of 1:5. The average mass deviation was less than 100 ppm. The matrix suppression mass cut off was m/z 700 Da. The following ion source parameters were used: ion source 1, 25.09 kV; ion source 2, 23.80 kV. Other settings for MALDI-TOF MS analysis were as follows: pulsed ion extraction, 260 ns and lens, 6.40 kV. FlexControl 3.4 software (Bruker Daltonics, Bremen, Germany) was applied for the acquisition and processing of the spectra. Each sample was analyzed in three repetitions. Inter-day and intra-day reproducibility of the applied procedure was evaluated in our previous study [22].

### nanoLC-MALDI-TOF-TOF MS/MS identification of discriminative peaks

The sample was prepared with ZipTip technique. The obtained eluent was further subjected to nano-LC separation using: nanoflow HPLC set (EASY-nano LC II, Bruker Daltonics, Germany) and fraction collector (Proteineer-fc II, Bruker Daltonics, Germany). The nano-LC system consisted of a trap column, NS-MP-10 BioSphere C18, (20 mm × 100 μm I.D., particle size 5 μm, pore size 120 Å) (NanoSeparations, Nieuwkoop, the Netherlands) and Thermo Scientific Acclaim PepMap 100 column C18 (150 mm × 75 μm I.D., particle size 3 μm, pore size 100 Å) (Thermo Scientific: Sunnyvale, CA, USA). Linear gradient was 2%–50% of ACN during 96 min. Two mobile phases were used: mobile phase A (0.05% TFA in water) and mobile phase B (0.05% TFA 90% ACN). The volume of injected sample eluent was 4 μL. The separation was performed with a flow rate 300 nL/min. A total of 384 fractions, 80 nL each, were obtained. Each eluent was automatically mixed with 420 nL of matrix solution that was prepared by mixing 36 μL of HCCA saturated solution of 0.1% TFA and ACN (90:10 *v*/v), 784 μL ACN and 0.1% TFA (95:5 *v*/v), 8 μL of 10% TFA and 8 μL of 100 mM ammonium phosphate monobasic and spotted onto the MALDI target (AnchorChip 800 μm) using a fraction collector. The system was controlled by HyStar 3.2 software (Bruker Daltonics, Germany). MALDI-TOF/TOF mass spectrometer (UltrafleXtreme, Bruker Daltonics, Germany) operated in a reflector mode was used in further analysis of the sample. The MS spectra were externally calibrated using Peptide Calibration Standard mixture (Bruker Daltonics, Germany). A list of precursor peaks was obtained using WARP-LC software (Bruker Daltonics, Germany). The chosen discriminative m/z were analyzed with MS/MS mode for protein identification. The parameters for MS and MS/MS mode were described in our previous study [22]. FlexControl 3.4 software (Bruker Daltonics, Germany) was applied for the acquisition of spectra. Processing and evaluation of the data was achieved using FlexAnalysis 3.4 (Bruker Daltonics, Germany). BioTools 3.2 (Bruker Daltonics, Germany) was used to perform protein database searches. Proteins were identified using the SwissProt database and Mascot 2.4.1 search engine (Matrix Science, London, UK) with taxonomical restriction to “*Homo sapiens*”. The following general protein search parameters were used: precursor-ion mass tolerance ±50 ppm; fragment-ion mass tolerance ±0.7 Da; no enzyme; monoisotopic mass; peptide charge +1.

### Data analysis

Data analysis of each spectrum was performed with ClinProTools version 3.0 software (Bruker Daltonics, Germany). In order to let the software group all analyzed sample replicates into one biological replicate, spectra grouping function was applied. This option provided improved measurement quality. Before any analysis or spectra processing, the multiple measurements were averaged. Further steps were processed upon one averaged spectrum per sample. Comparison of the obtained data was achieved through a standard workflow. Each spectrum was first normalized to the total ion current (TIC) and recalibrated with the prominent common m/z values. “Top hat” baseline subtraction with the minimum baseline width set to 10% was used to remove broad structures. Spectra were also smoothed and processed in the mass range of 1000–10,000 Da. The signal-to-noise ratio was greater than or equal to 5. Peak picking and average peak calculation procedures were used. A total average spectrum was calculated from the preprocessed spectra. Averaging of the spectra allowed us to improve the signal to noise during peak picking procedure. Due to average peak list calculation, small peaks that might be missed on a single spectrum, were included in the overall profile. All reproducible peaks were detected according to this procedure.

**Fig. 1:**
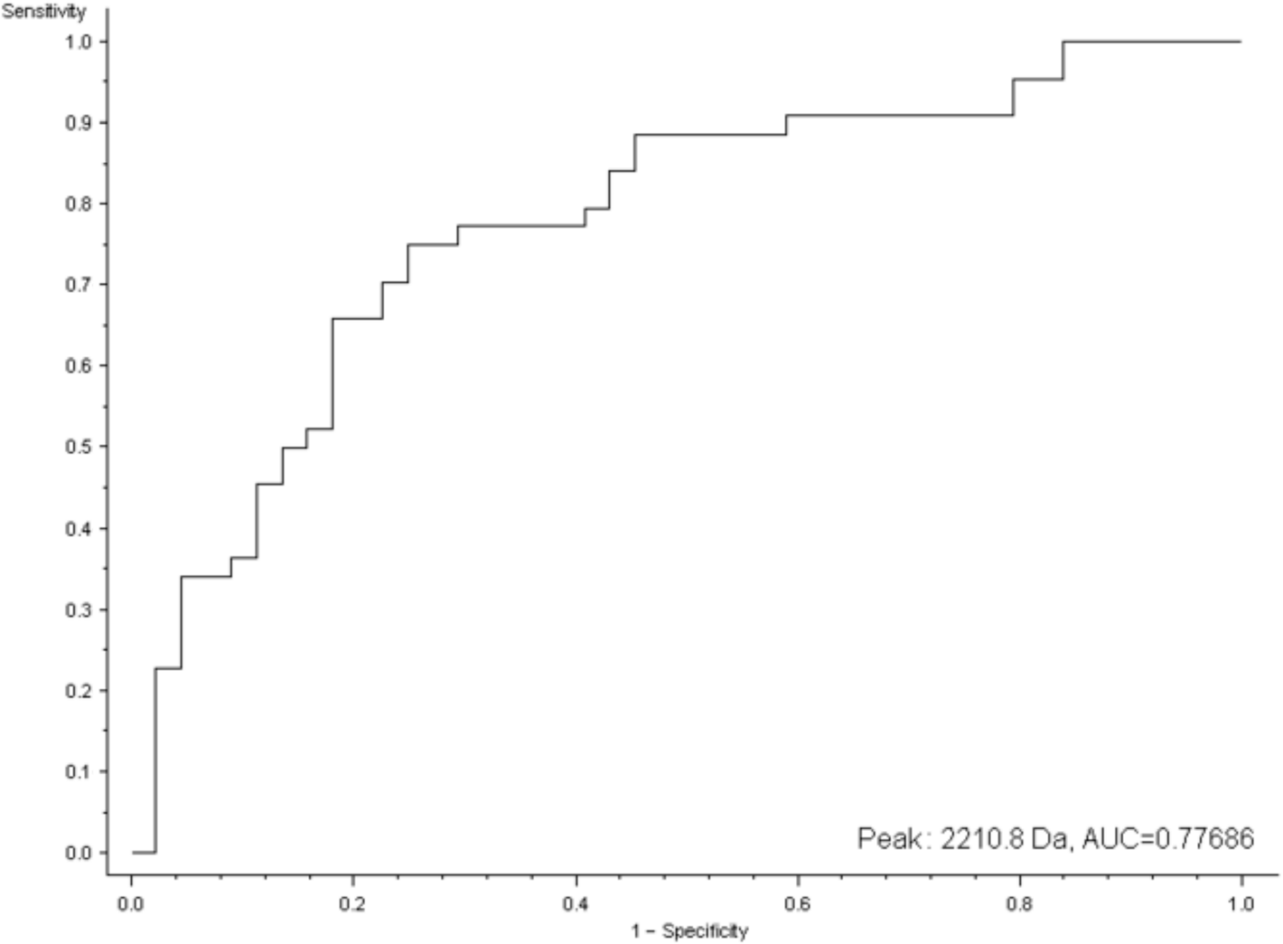
Receiver operating characteristic (ROC) curve representing sensitivity and specificity of m/z peak 2210.8 Da. Area under the ROC curve (AUC) is 0.78

Comparisons between patients with OC and healthy individuals were evaluated with Wilcoxon test. Statistical significance was attained when *p*-value was ≤0.02. All *p*-values were internally corrected with the Benjamini-Hochberg algorithm. Evaluation of the discrimination ability of each peak was achieved by calculating receiver operating characteristic (ROC) curve and the area under the ROC curve (AUC) (Fig. 1). Chemometric algorithms: supervised neural network (SNN), genetic algorithm (GA), and quick classifier (QC) were used for model analysis and selection of peptide/protein peak clusters. Each model indicated a combination of the differentiating peaks. The studied groups were randomly subdivided into a training set (containing 33 ovarian cancer patients and 33 healthy controls) and a test set (containing 11 ovarian cancer patients and 12 healthy controls). The use of these two sets allowed for testing robustness of the obtained models. For the training set two parameters, 20% leave one out cross validation and recognition capability, were calculated. For the model with the best performance of these two indicators, an external validation using the test set was calculated. The values of sensitivity and specificity were used to define discriminative ability of the model. Peaks that indicated the best discrimination between the studied groups were further identified as fragments of defined proteins.

## Results

Eighty nine serum samples derived from ovarian cancer patients (*n* = 44) and healthy individuals (*n* = 45) were pretreated with ZipTips and analyzed in triplicate by MALDI-TOF MS. The reproducibility and reliability of the used methodology were evaluated and described in our previous report by calculating inter-day and intra-day variability [22]. The average coefficient of variation (CV) for inter-day study was 20.0% and for intra-day study it was 6.9%. The combination of ZipTips technology and MALDI-TOF MS analysis allowed us to generate a total of 170 spectral components (m/z unique peaks) from the serum samples. Univariate statistical analysis based on Wilcoxon test identified 98 peaks as significantly different between the studied groups. Moreover, discriminatory power of the obtained peaks was further analyzed by calculating the ROC curve, which represents a graphical relation between sensitivity and specificity (Fig. 1). Based on univariate tests, discriminative ability of the detected peaks was examined (Additional file 2, Table S2).

Panels of multiple disease markers manifest more powerful discriminative abilities than a single uncorrelated marker. Therefore, three mathematical algorithms (SNN, GA and QC) were used in order to generate prediction models based on the training set with randomly selected samples (cancer patients *n* = 33 and healthy controls *n* = 33). Combinations of peaks used by these algorithms are shown in the Table 2. Six peaks (m/z) are present in more than one model. However, only the peak of 2082.75 Da occurs in all three discriminatory panels. Two parameters (recognition capability and cross validation) were calculated for all used discriminative models (Table 3). Cross validation of the established models reached 63.64% (SNN), 54.55% (GA) and 68.18% (QC), while recognition capability rates were 80.30% (SNN), 93.94% (GA) and 72.72% (QC). External validation was proceeded using independent data set (cancer patients *n* = 11 and healthy controls *n* = 12). The highest values of sensitivity (71.00%) and specificity (68.60%) were associated with SNN (Table 3). This model was composed of 25 different peaks. According to the univariate tests (Wilcoxon test and ROC curve) 10 of them revealed statistically significant variation between studied groups with *p*-values <0.02 and AUC in the range 0.67–0.78.

**Table 2:** Combinations of peaks (m/z) used in calculated algorithms (SNN, QC and GA)

SNN (Da)	QC (Da)	GA (Da)
1364.60	1466.76	1207.36
1419.84	1945.40	1229.32
1435.84	2082.75	1419.84
1505.28	2116.16	1509.45
1509.45	2604.30	1520.10
1538.10	3158.79	1639.82
1741.40	3507.55	1888.54
1897.69	4075.32	2082.75
1945.40	4112.79	4249.16
2023.30	4151.55	5753.62
2082.75	4209.96	
2116.16	4231.58	
2210.84	4249.16	
2453.10	4268.99	
2770.01	4282.73	
2863.35	4644.22	
3158.79	4663.57	
3192.56	4680.02	
3263.59	4712.28	
3284.04	4755.77	
3302.35	5065.16	
6631.04	6376.98	
7692.26	6395.79	
7767.39	6585.81	
8602.82

**Table 3:** Results of recognition capability, cross validation, sensitivity and specificity for discriminative models (SNN, QC and GA)

	SNN	QC	GA
Recognition capability (%)	80.30	72.73	93.94
Cross validation (%)	63.64	68.18	54.55
Sensitivity (%)	71.00	77.40	87.10
Specificity (%)	68.60	51.40	48.60

In order to identify the peaks that, according to statistical analyses, had the highest diagnostic efficacy (linear positive mode m/z 1505.24; 1945.38; 2023.17; 2082.73; 2116.08; 2210.80; 3158.75; 6560.82; 7567.69 and 7830.60 Da) (Table 4), serum samples were pretreated with ZipTips and examined using tandem mass spectrometry nano-LC-MALDI-TOF/TOF-MS/MS. The spectra were analyzed in the mass range of 700–3500 Da in the reflector mode, which requires using sufficient resolution. It enables proper baseline separation of the analyzed peaks and highly accurate determination of their mass [15]. Therefore, discriminative peaks with mass of m/z: 6560.82; 7567.69 and 7830.60 were not detected. The MS/MS analysis of precursor ions m/z 1504.8231 and 2021.1246 resulted in identification of Complement C3 protein (CO3_HUMAN) based on the peptide sequence G.SPMYSIITPNILR.L and R.SSKITHRIHWESASLLR.S, respectively, with significant hit in the Mascot search. Another discriminative peak, precursor ion m/z 1943.9257, was identified according to the MS/MS fragmentation as sequence H.NLGHGHKHERDQGHGHQ.R with high score in the Mascot database to Kininogen-1 protein (KNG1_HUMAN). Identification of both precursors m/z 2083.0695 and 3156.5613 allowed for obtaining the sequences: P.GVLSSRQLGLPGPPDVPDHAA.Y and R.NVHSGSTFFKYYLQGAKIPKPEASFSPR.R, respectively, with significant hit in the Mascot database to Inter-alpha-trypsin inhibitor heavy chain H4 (ITIH4_HUMAN). The fragmentation of signal m/z 2210.0565 allowed us to identify the following peptide sequence: G.ISPFHEHAEVVFTANDSGPR.R. It gave a significant score in the Mascot search to transthyretin protein (TTHY_HUMAN). Despite our efforts to extend the time of nano-LC gradient to 150 min, the obtained MS/MS spectrum of signal m/z 2016.8865 was contaminated with other neighborhood fragments or contained some unknown modification. For these reasons, its identification failed. For the hypothetical peptide sequence: TSSTSYNRGDSTFESKSY of the m/z 2016.8865, no results in the Mascot search were obtained. However, homology to fibrinogen alpha chain isoform alpha-E preproprotein (FIBA_HUMAN) was shown according to the MS-BLAST database. Therefore, the identification of this peak would require further analysis.

**Table 4:** The most discriminative peaks (m/z signals) according to Wilcoxon test (*p*-values), ROC curve (AUC) and mathematical model (SNN) with their identification

Mass (m/z)	*p*-value	AUC	Peptide sequence	Identification	Ref.
1505.24	0.00937	0.676	G.SPMYSIITPNILR.L	CO3_HUMAN	[22, 24, 34, 35]
1945.38	0.00290	0.725	H.NLGHGHKHERDQGHGHQ.R	KNG1_HUMAN	[36–40]
2023.17	0.01280	0.667	R.SSKITHRIHWESASLLR.S	CO3_HUMAN	[22, 24, 34, 35]
2082.73	0.00056	0.767	P.GVLSSRQLGLPGPPDVPDHAA.Y	ITIH4_HUMAN	[37, 41–45]
2116.08	0.00171	0.736	Hypothetical TSSTSYNRGDSTFESKSY	Hypothetical FIBA_HUMAN	-
2210.80	0.00056	0.777	G.ISPFHEHAEVVFTANDSGPR.R	TTHY_HUMAN	[9, 46–48]
3158.75	0.00171	0.738	R.NVHSGSTFFKYYLQGAKIPKPEASFSPR.R	ITIH4_HUMAN	[37, 41–45]
6560.82	0.00689	0.680	-	-	-
7567.69	0.01180	0.671	-	-	-
7830.60	0.00451	0.700	-	-	-

## Discussion

OC is the most deadly gynecological cancer [23]. Due to scarceness of symptoms and lack of effective screening tests, this disease remains undetected until advanced stages. Therefore, in an attempt to discover high sensitivity biomarkers, a rapid development of novel approaches is observed. In the literature, there are several studies focusing on plasma and serum proteomic patterns of ovarian cancer obtained by MALDI-TOF MS. In order to detect low abundance proteins and peptides, biomarker enrichment kits [24], immunodepletion [25] and magnetic beads [26] have been applied in OC studies. Due to significant impact of sample pretreatment on the MS spectra, the discriminative peaks proposed as candidates for OC biomarkers depend on efficiency of the enrichment strategy. Thus, in the present study a solid phase extraction technology - micropipette tips ZipTips - was proposed as a depletion method with the aim of low molecular peptide/protein characterization of the serum protein-peptide profiles of the OC. The applied methodology constitutes an objective tool for identification of the OC indicators, which may contribute to understanding the pathological processes and may facilitate the development of both novel diagnostic tools and molecular targeted therapies.

The serum proteomic patterns of OC were obtained by MALDI-TOF MS. All spectra were analyzed using univariate tests including ROC curve and Wilcoxon test. However, according to the literature typing only single disease biomarker is not sufficient and multi-component combinations may lead to the design of new diagnostic tools characterized by significant sensitivity and specificity [27, 28]. Therefore, in this study discrimination models were calculated using three different mathematical algorithms. Differences in their combinations of components are caused by various calculation mechanisms [29]. SNN allows for an identification of the most characteristic spectra for all the studied groups. They are called prototypes and reflect prototypical samples of each class [22, 30]. GA is based on natural evolution, which enables selection of the most important variables. A cost function leads to significant class selection [29]. QC, a univariate sorting algorithm, calculates average peak areas for each class and stores them together with other data like *p*-values at defined peak positions. Prediction models are created based on weighted average derived from all peaks [30].

For the calculated models two parameters (cross validation and recognition capability) were determined. Discriminatory models tend to achieve better results on data on the basis of which they were originally constructed than on data derived from a new set of samples [31]. Thus, cross validation might be insufficient to assess the power of the obtained multi-component panel. For that reason, external validation seems an important step in defining accuracy of a created model [32]. Nevertheless, some clinical studies focus only on the internal validation and leave behind the need of the external validation [31]. In this study, independent test sets were used to evaluate robustness of the prediction models. Due to difficulties in selecting a model with the best diagnostic efficacy based on recognition capability and cross validation, external validation was performed for all three classification algorithms (Table 3). The best differentiating capabilities and satisfactory values of sensitivity (71.00%) and specificity (68.60%) were associated with SNN.

Protein-peptide profiling studies based on MALDI-TOF MS analyses are often limited to a list of the most discriminative m/z peaks as potential disease indicators [15, 25, 26]. Nevertheless, the identification step is crucial for understanding the pathological processes that occur during cancer development and it should not be omitted. However, protein identification using MALDI-TOF without a digestion step might be challenging. Thus, the subject literature contains reports on combining MALDI-TOF profiling with other mass spectrometry platforms [24, 33]. This study proposes a novel approach that enables protein-peptide profiling as well as identification of clusters of ions with diagnostic capability using tandem mass spectrometry nano-LC-MALDI-TOF/TOF-MS/MS.

Application of developed strategy allowed for the identification of four potential OC serum biomarkers (complement C3, kininogen-1, inter-alpha-trypsin inhibitor heavy chain H4, and transthyretin). Complement C3 plays a key role in both immunological and inflammatory processes. Recent findings suggest that it may promote tumor growth, angiogenesis, cellular proliferation and regeneration [34]. Thus, a new concept of cancer treatment based on blocking the complement system was proposed [35]. Moreover, a number of studies reported that patients with cancer (including OC) produce altered levels of complement C3 as compared with healthy subjects [22, 24]. It might be caused by an inflammatory response to the tumor development. However, this marker should be further validated with the use of controls from inflammatory conditions.

Another identified protein – kininogen-1 takes part in blood coagulation and in the kinin-kallikrein system. It shows antiangiogenic properties and it also inhibits proliferation of endothelial cells. The role of this protein in cancer development might be associated with survival of the cancer cells [36, 37]. Changes in the levels of kininogen-1 were observed in urine samples of OC patients [37]. Its expression was altered in the serum and plasma in patients with proliferative vitreoretinopathy [38] and colorectal cancer [36]. Moreover, other diseases like interstitial cystitis [39] or IgA nephropathy [40] are also related to non-standard urine concentrations of kininogen-1.

Protein also identified as potential OC marker is ITIH4, which belongs to the inter-alpha-trypsin inhibitor (ITI) family and it is an acute-phase reactant [41]. There are a few reports that proposed this protein as an OC marker [37, 42]. Changes in the levels of ITIH4-derived peptides were also observed in urine of early prostate cancer patients [43] and in the serum of breast cancer patients [44] and gastric adenocarcinoma patients [41]. A correlation was also suggested between different fragmentation of ITIH4 and disease conditions [45].

The last protein proposed as discriminatory marker of OC is transthyretin. Transthyretin plays an essential role in a transport of thyroxine and tri-iodothyronine. It also takes part in the transfer of retinol. Differences in the expression of transthyretin during OC development were already reported [46, 47]. Moreover, changes in the cellular retinol binding protein levels in OC patients were observed [48]. What is worth emphasizing, transthyretin is one of the five biomarkers the concentrations of which are measured in OVA1 multivariate index assay [9].

The applied methodology allowed us to identify four different serum proteins (Table 4) with an essential role in OC development, according to the literature. Complement C3, inter-alpha-trypsin inhibitor heavy chain H4 and transthyretin were also identified using a combination of carrier protein-bound affinity enrichment strategy with MALDI-TOF MS to characterize OC samples, which is in agreement with our findings [24]. Unfortunately, other OC studies based on MALDI-TOF MS profiling lack the identification step [25, 26]. It is unknown whether other depletion methods are capable of detecting relevant proteins.

Naturally, MALDI-TOF MS is a very sensitive technique of qualitative analysis [49] that should be complemented with a quantitative approach to confirm the results of peptide-protein profiling. Thus, the clinical utility of complement C3, kininogen-1, inter-alpha-trypsin inhibitor heavy chain H4 and transthyretin should be examined by quantitative analysis in a larger set of samples. Moreover, further studies are planned to identify the remaining discriminative peaks since they might extend our knowledge on the pathological processes that occur during OC.

## Conclusions

To conclude, proteomic profiling of serum samples based on the solid phase extraction enrichment technology coupled with MALDI-TOF MS demonstrated differences in the serum protein expression in patients with OC compared with the healthy control group. The SNN classification algorithm yielded a discriminative model characterized by significant sensitivity (71%) and specificity (68.6%) in the external validation. The novel approach, which enabled protein-peptide profiling as well as identification of four potential OC biomarkers (complement C3, kininogen-1, inter-alpha-trypsin inhibitor heavy chain H4 and transthyretin) using MALDI-TOF MS, may contribute to the creation of new effective multi-component diagnostic tools. Additionally, a panel of discriminative proteins could provide an explanation of complex signaling and functional networks associated with this multifactorial disease.

## Additional files

Additional file 1: Table S1. Study group characterization according to histopathological type and FIGO stage at diagnosis. (DOCX 12 kb)

Additional file 2: Table S2. Masses (m/z) and intensities of the peaks with the highest values of the univariate statistical tests: Wilcoxon test and the ROC curve (*p*-value <0.05; AUC > 0.7). (DOCX 14 kb)

## Abbreviations

ACN: acetonitrile
AUC: area under the ROC curve
CA125: cancer antigen 125
CO3_HUMAN: Complement C3 protein
CV: coefficient of variation
FDA: Food and Drug Administration
FIBA_HUMAN: fibrinogen alpha chain isoform alpha-E preproprotein
GA: genetic algorithm
HCCA: α-cyano-4-hydroxycinnamic acid
HE4: human epididymis protein 4
ITIH4_HUMAN: Inter-alpha-trypsin inhibitor heavy chain H4
KNG1_HUMAN: Kininogen-1 protein
MALDI-TOF-MS: Matrix-Assisted Laser Desorption/Ionization Time-Of-Flight Mass Spectrometry
MIA: multivariate index assay
nano-LC: nano-liquid chromatography
OC: Ovarian carcinoma
QC: quick classifier
ROC: receiver operating characteristic
ROMA: Risk of Ovarian Malignancy Algorithm
SNN: supervised neural network
TFA: trifluoroacetic acid
TIC: total ion current
TTHY_HUMAN: transthyretin protein
