# Supplementary material for: MALDI-TOF-MS analysis in discovery and identification of serum proteomic patterns of ovarian cancer
Source: BMC Cancer. 2017 Jul 6;17:472. doi: 10.1186/s12885-017-3467-2 (PMC5501370; doi:10.1186/s12885-017-3467-2)
Supplement: Supplementary file 1 — Study group characterization according to histopathological type and FIGO stage at diagnosis. (DOCX 12 kb) [file 12885_2017_3467_MOESM1_ESM.docx]

**Additional file 1**

**Table S1**

Study group characterization according to histopathological type and FIGO stage at diagnosis.

|  | **Number of samples** | |
| --- | --- | --- |
| **Histopathological type** | **OC training set** | **OC test set** |
| Serous | 10 | 7 |
| Endometrioid | 3 | 2 |
| Mucinous | 1 | 0 |
| Clear cell | 3 | 0 |
| Undifferentiated | 9 | 1 |
| Non identified | 2 | 1 |
| **FIGO stage at diagnosis** | | |
| I | 9 | 1 |
| II | 2 | 0 |
| III | 16 | 10 |
| IV | 1 | 0 |
