# Supplementary material for: MALDI-TOF-MS analysis in discovery and identification of serum proteomic patterns of ovarian cancer
Source: BMC Cancer. 2017 Jul 6;17:472. doi: 10.1186/s12885-017-3467-2 (PMC5501370; doi:10.1186/s12885-017-3467-2)
Supplement: Supplementary file 2 — Masses (m/z) and intensities of the peaks with the highest values of the univariate statistical tests: Wilcoxon test and the ROC curve (p-value <0.05; AUC > 0.7). (DOCX 14 kb) [file 12885_2017_3467_MOESM2_ESM.docx]

**Additional file 2**

**Table S2**

Masses (m/z) and intensities of the peaks with the highest values of the univariate statistical tests: Wilcoxon test and the ROC curve (p-value < 0.05; AUC > 0.7).

| **Mass (m/z)**  **(Da)** | **AUC** | **p-value** | **Peak intensity average of the OC group** | **Peak intensity average of the control group** |
| --- | --- | --- | --- | --- |
| 1466.75 | 0.713 | 0.00360 | 196.15 | 116.56 |
| 1488.69 | 0.706 | 0.00431 | 34.27 | 20.41 |
| 1945.38 | 0.725 | 0.00290 | 2.66 | 7.60 |
| 2082.75 | 0.767 | 0.00056 | 1.36 | 3.40 |
| 2116.08 | 0.736 | 0.00171 | 1.02 | 2.50 |
| 2210.80 | 0.777 | 0.00056 | 0.76 | 3.02 |
| 2604.33 | 0.762 | 0.00056 | 1.33 | 3.68 |
| 3158.75 | 0.738 | 0.00171 | 0.86 | 2.08 |
| 3814.27 | 0.702 | 0.00437 | 0.20 | 0.45 |
| 3955.21 | 0.738 | 0.00171 | 0.57 | 1.39 |
| 4075.27 | 0.710 | 0.00360 | 0.38 | 0.87 |
| 4112.61 | 0.702 | 0.00437 | 0.35 | 0.67 |
| 4209.95 | 0.726 | 0.00307 | 5.68 | 12.20 |
| 4231.53 | 0.712 | 0.00360 | 1.00 | 1.93 |
| 4249.11 | 0.712 | 0.00360 | 0.78 | 1.57 |
| 4268.87 | 0.735 | 0.00171 | 0.62 | 1.37 |
| 4282.60 | 0.763 | 0.00056 | 0.41 | 1.48 |
| 4644.14 | 0.715 | 0.00347 | 0.23 | 0.44 |
| 4663.50 | 0.723 | 0.00297 | 0.15 | 0.25 |
| 4679.48 | 0.709 | 0.00395 | 0.15 | 0.26 |
| 4697.17 | 0.705 | 0.00431 | 0.12 | 0.21 |
| 4711.30 | 0.717 | 0.00337 | 0.13 | 0.27 |
| 4755.70 | 0.740 | 0.00171 | 0.12 | 0.25 |
| 5004.65 | 0.724 | 0.00297 | 0.39 | 2.68 |
| 5025.56 | 0.718 | 0.00333 | 0.15 | 0.48 |
| 5044.40 | 0.743 | 0.00171 | 0.15 | 0.51 |
| 5065.16 | 0.735 | 0.00171 | 0.15 | 0.41 |
| 5081.56 | 0.707 | 0.00437 | 0.12 | 0.35 |
| 5103.57 | 0.703 | 0.00437 | 0.10 | 0.19 |
| 5160.64 | 0.708 | 0.00407 | 0.08 | 0.50 |
| 6377.14 | 0.711 | 0.00360 | 0.05 | 0.08 |
| 6453.90 | 0.702 | 0.00437 | 0.06 | 0.10 |
| 6472.43 | 0.702 | 0.00437 | 0.06 | 0.10 |
| 6514.86 | 0.705 | 0.00437 | 0.04 | 0.07 |
| 6587.92 | 0.711 | 0.00360 | 0.05 | 0.08 |
| 6651.81 | 0.720 | 0.00331 | 0.08 | 0.16 |
| 6670.30 | 0.716 | 0.00342 | 0.08 | 0.16 |
| 6710.56 | 0.701 | 0.00439 | 0.05 | 0.08 |
